# A Novel Bacteriophage with Broad Host Range against Clostridioides difficile Ribotype 078 Supports SlpA as the Likely Phage Receptor

**DOI:** 10.1128/spectrum.02295-21

**Published:** 2022-02-02

**Authors:** M. J. Whittle, T. W. Bilverstone, R. J. van Esveld, A. C. Lücke, M. M. Lister, S. A. Kuehne, N. P. Minton

**Affiliations:** a Clostridia Research Group, BBSRC/EPSRC Synthetic Biology Research Centre (SBRC), School of Life Sciences, Biodiscovery Institute, The University of Nottingham, Nottingham, United Kingdom; b NIHR Nottingham Biomedical Research Centre, Nottingham University Hospitals NHS Trust and The University of Nottingham, Nottingham, United Kingdom; c Faculty of Medicine, Leiden University Medical Centre, the Netherlands; d Hannover Medical School, Hannover, Germany; University of Nebraska-Lincoln

**Keywords:** bacteriophage, phage therapy, *Clostridioides difficile* (*Clostridium difficile*), S-layer, SlpA, phage receptor

## Abstract

Bacteriophages represent a promising option for the treatment of Clostridioides difficile (formerly Clostridium difficile) infection (CDI), which at present relies on conventional antibiotic therapy. The specificity of bacteriophages should prevent dysbiosis of the colonic microbiota associated with antibiotic treatment of CDI. While numerous phages have been isolated, none have been characterized with broad host range activity toward PCR ribotype (RT) 078 strains, despite their relevance to medicine and agriculture. In this study, we isolated four novel C. difficile myoviruses: ΦCD08011, ΦCD418, ΦCD1801, and ΦCD2301. Their characterization revealed that each was comparable with other C. difficile phages described in the literature, with the exception of ΦCD1801, which exhibited broad host range activity toward RT 078, infecting 15/16 (93.8%) of the isolates tested. In order for wild-type phages to be exploited in the effective treatment of CDI, an optimal phage cocktail must be assembled that provides broad coverage against all C. difficile RTs. We conducted experiments to support previous findings suggesting that SlpA, a constituent of the C. difficile surface layer (S-layer) is the likely phage receptor. Through interpretation of phage-binding assays, our data suggested that ΦCD1801 could bind to an RT 012 strain only in the presence of a plasmid-borne S-layer cassette corresponding to the *slpA* allele found in RT 078. Armed with this information, efforts should be directed toward the isolation of phages with broad host range activity toward defined S-layer cassette types, which could form the basis of an effective phage cocktail for the treatment of CDI.

**IMPORTANCE** Research into phage therapy has seen a resurgence in recent years owing to growing concerns regarding antimicrobial resistance. Phage research for potential therapy against Clostridioides difficile infection (CDI) is in its infancy, where an optimal “one size fits all” phage cocktail is yet to be derived. The pursuit thus far has aimed to find phages with the broadest possible host range. However, for C. difficile strains belonging to certain PCR ribotypes (RTs), in particular RT 078, phages with broad host range activity are yet to be discovered. In this study, we isolate four novel myoviruses, including ΦCD1801, which exerts the broadest host range activity toward RT 078 reported in the literature. Through the application of ΦCD1801 to phage-binding assays, we provide data to support the prior notion that SlpA represents the likely phage receptor on the bacterial cell surface. Our finding directs research attention toward the isolation of phages with activity toward strains possessing defined S-layer cassette types.

## INTRODUCTION

Clostridioides difficile (formerly Clostridium difficile [1]) is the leading cause of hospital-associated diarrhea in the developed world, responsible for up to 29,000 deaths per annum in the United States (2). C. difficile infection (CDI) ensues from dysbiosis of the gut microbiota, in response to broad-spectrum antibiotic treatment (3). Up to 65% of patients suffer recurrent infection or relapse following treatment of CDI with metronidazole or vancomycin (4). This phenomenon is a consequence of the spore-forming nature of C. difficile, concomitant with the reduced-diversity microbiota following sustained antibiotic therapy (5). This unfortunate chain of circumstance, whereby the antibiotic for the treatment of CDI is also the predisposing risk factor for its contraction, calls for a more directed approach to combatting this infection, an approach that minimally disrupts the diversity of the gut microbiota.

Bacteriophages (phages) are generally considered narrow host range viruses, where host specificity can be observed at the genus, species, or subspecies level (6). Consequently, bacteriophages might represent an appropriate narrow-spectrum therapy for the treatment of CDI. To date, many phages infecting C. difficile have been characterized, all of which are temperate *Myoviridae*/*Siphoviridae* belonging to the *Caudovirales* order of phages. However, no phage has been described with broad host range activity toward PCR ribotype 078 (RT 078), strains of which are of considerable clinical and agricultural relevance (7).

While the efficacy of single-phage therapy has little remedial effect *in vivo*, combinatorial therapy has demonstrated some merit. Therein, a cocktail of phages was able to delay the time to endpoint by almost 100% in hamsters infected with one strain of C. difficile (8). These promising data warrant further study into optimal phage cocktail combinations.

The efforts toward combinatorial phage cocktails would be considerably boosted had the phage receptor on the surface of the bacterial cell been confirmed. Thus far, two research articles have provided evidence suggesting that the surface layer (S-layer) constituent, SlpA, represents the likely phage receptor candidate for C. difficile phage infection (9, 10). Kirk and colleagues demonstrated that the R-type bacteriocin Avidocin-CD, which structurally mimics a myovirus devoid of a nucleic acid-containing capsid, could be retargeted toward different surface layer cassette types (SLCTs) by inclusion of receptor-binding proteins (RBPs) from C. difficile phages associated with specific SLCTs (9). Doing so confirmed the role of SlpA as a receptor for Avidocin-CD infection. The ability for phage RBPs to alter Avidocin-CD sensitivity in an SLCT-dependent manner provides strong evidence that SlpA represents the receptor for phage infection. Soon after, Phothichaisri and colleagues provided data suggestive of phage particles binding to SlpA, since the inclusion of phage particles to SlpA samples led to retardation compared with samples lacking phage particles when run by SDS-PAGE (10). We sought to build upon the above-mentioned findings by probing the ability for exogenous SlpA to permit cross-ribotype binding of phages to C. difficile.

In this study, we isolated and characterized four novel bacteriophages, one of which, ΦCD1801, possessed broad host range activity against C. difficile PCR ribotype 078 (RT 078). We demonstrate that plasmids harboring the *slpA* allele corresponding to the RT 078 S-layer cassette (H2/6) and SLCT 6 permitted cross-ribotype binding of ΦCD1801 to the RT 012 strain CD630 (SLCT 7). These data contribute to the wealth of existing evidence to suggest that SlpA represents the likely phage receptor for C. difficile.

## RESULTS AND DISCUSSION

### Isolation and visualization of four novel myoviruses.

We sought to isolate phages with infective capacity toward RT 078. RT 078 strains are often considered potentially hypervirulent (11). Indeed, strains possess the binary toxin genes (C. difficile transferase [CDT]), while their clinical presentation is comparable to that of the notorious RT 027 (12), the hypervirulent ribotype responsible for severe outbreaks across North America and Europe (13). To enhance our efforts, we obtained a library of clinical isolates from CDI-positive patients at the Queens Medical center (Nottingham, UK), which included eight novel RT 078 isolates (see Table S1 for novel strains described in this article). Using CD1801 as an isolation host, we were able to isolate ΦCD1801 from an anaerobic digester sample derived from the Stoke Bardolph sewage treatment plant (Nottinghamshire, UK). In parallel, we isolated ΦCD08011, ΦCD2301, and ΦCD522418 (hereafter referred to as ΦCD418) from RT 002, 014, and 023 hosts, respectively.

**FIG 1:**
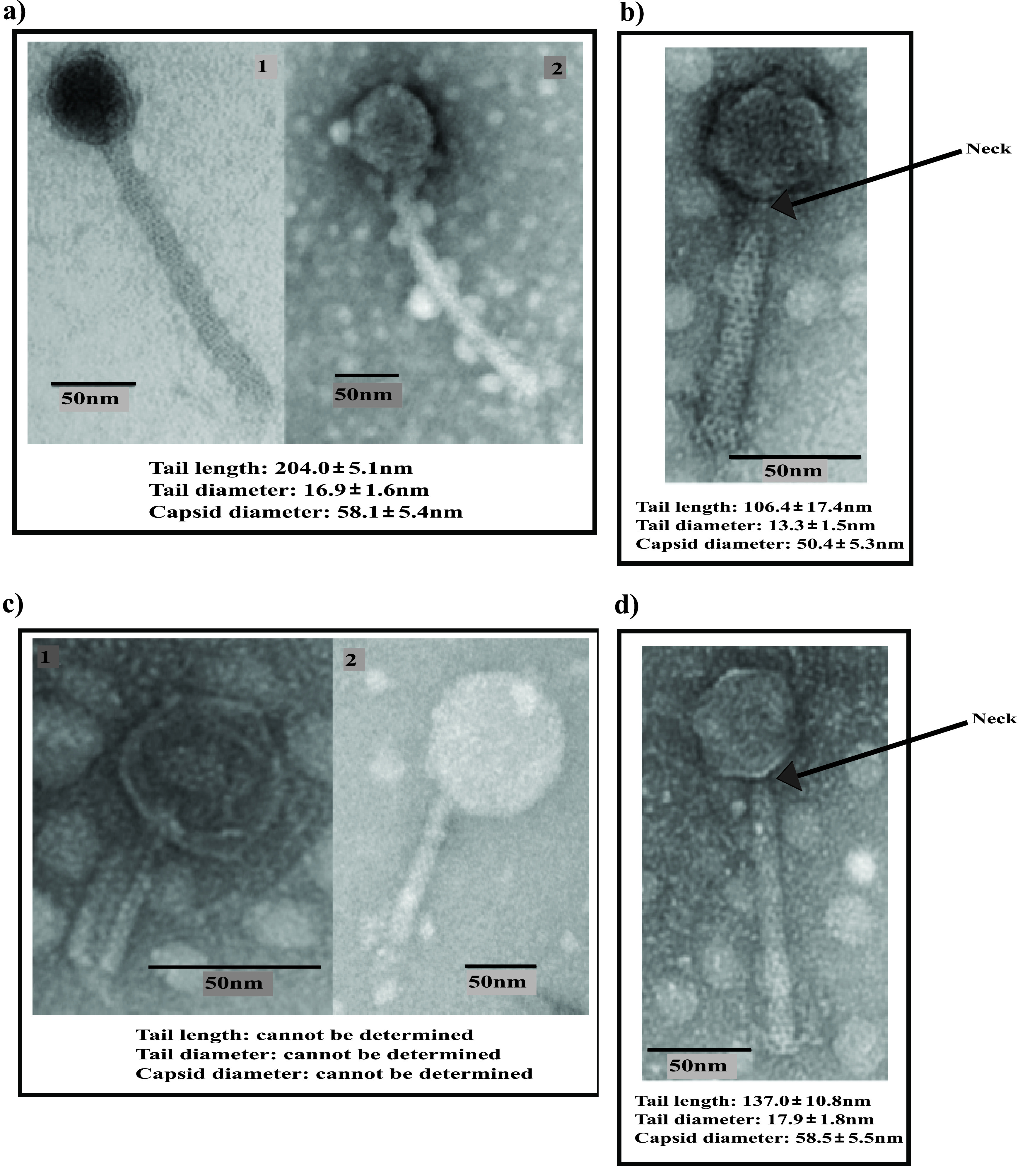
Phage particle morphology as visualized using TEM for (a) ΦCD418, (b) ΦCD2301, (c) ΦCD1801, and (d) ΦCD08011. Measurements represent the mean ± standard deviation (SD) of values of 5 individual phage particles.

Transmission electron microscopy (TEM) analysis revealed that ΦCD418, ΦCD2301, and ΦCD1801 possessed contractile tails (Fig. 1a to c), suggesting they belonged to the *Caudovirales* order of tailed phages and were, like most of the published C. difficile phages, myoviruses. Indeed, the tail and capsid measurements are in line with those previously reported for C. difficile myoviruses (14, 15). The imaging results were less clear for ΦCD08011, wherein, after multiple experiments, phage particles always appeared with contracted tails and empty capsids indicative of DNA release (Fig. 1d). In light of these issues, we are unable to definitively state that ΦCD08011 is a member of the *Myoviridae* family.

### Phage genome sequencing, annotation, and analysis.

**FIG 2:**
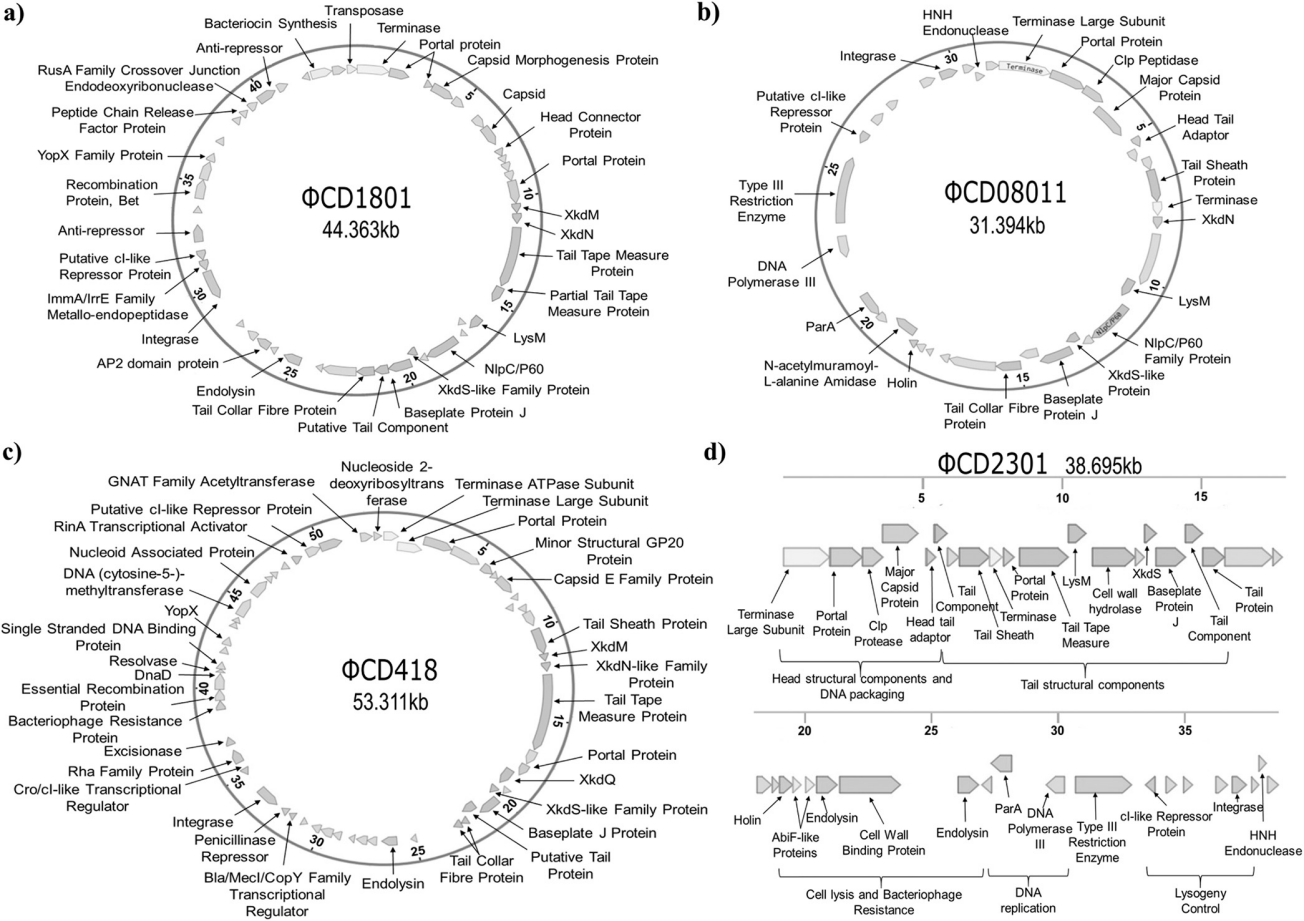
Graphical representation of phage genomes. (a) ΦCD1801, (b) ΦCD08011, (c) ΦCD418, and (d) ΦCD2301. Assembled using CLC Genomics Workbench and manually annotated using Artemis, BLAST, UniProt, and pfam. Genes of particular importance are discussed in the main body of text. Notably, the genomes contain integrase genes indicating their temperate nature. Proteins making up the capsids and tails, including the base plate proteins, were also identified.

Whole-genome sequencing of ΦCD1801 followed by *de novo* assembly revealed a 44,363-bp circular genome with a GC content of 28.87%. Artemis software predicted the genome to contain 50 open reading frames (ORFs), of which putative function could be assigned to 32. A graphical representation of the ΦCD1801 genome is provided in Fig. 2a. A lytic repressor protein (CD1801_gp35) was annotated by sequence alignment with repressor proteins derived from other published C. difficile phage sequences using the EMBOSS pairwise sequence-alignment tool (16). Doing so unveiled 100% amino acid similarity with the repressor protein in C. difficile phage ΦCD27 (17). A head connector protein (CD1801_gp8) was also located within the genome, further confirming the classification of ΦCD1801 as a myovirus. The genome was closed via PCR with primers annealing to the left and right flanks of the assembled contig. Primer sequences for the closure of phage genomes are provided in Table S2.

Using the above-mentioned analyses, closed genome maps were generated for ΦCD08011 and ΦCD418 with genome lengths of 31,394 and 53,311 bp, respectively (Fig. 2b and c). Both genomes comprised double-stranded DNA (dsDNA) with GC contents of 29.81% (ΦCD08011) and 29.07% (ΦCD418). A total of 35 ORFs were detected for ΦCD08011, of which 23 were assigned putative function, compared with 35 putative functional protein-coding genes from a total of 58 for ΦCD418.

It was not possible to generate a closed genome for ΦCD2301. While the 38,695-bp dsDNA genome could be assembled into one single contig, it was not possible to close the genome via PCR at the left and right flanks of the assembled reads, despite repeated attempts. As such, we present this genome as a linear fragment (Fig. 2d). A total of 39 ORFS were detected for ΦCD2301, of which 27 could be assigned putative function.

*In silico* analysis revealed the presence of integrase genes in the genomes of all four phages, corresponding to ΦCD1801_gp33, ΦCD08011_gp33, ΦCD418_gp36, and ΦCD2301_gp37. Their presence suggests that all four are, in common with all previously isolated C. difficile phage, temperate in nature. This conclusion was confirmed by the subsequent isolation of C. difficile lysogens for each phage.

Annotated phage genomes were submitted to GenBank as a BankIt submission. The accession numbers for each genome are as follows: ΦCD1801 (MW512570), ΦCD08011 (MW512572), ΦCD418 (MW512573), and ΦCD2301 (MW512571).

### Phage host range testing.

Thus far, we had isolated four myovirus phages that individually infect at least one RT 001, 014, 023, and 078 isolate. Genomic and phenotypic analysis thereof suggests that the novel phages are comparable to other phages reported for C. difficile. Until then, there had been few phages characterized with infective capacity for RT 078, most of which demonstrated very narrow host ranges within the ribotype. For example, when a panel of seven phages were screened for their ability to infect eight RT 078 isolates, seven were unable to infect any RT 078 strain, while one phage was able to infect three strains (8).

**FIG 3:**
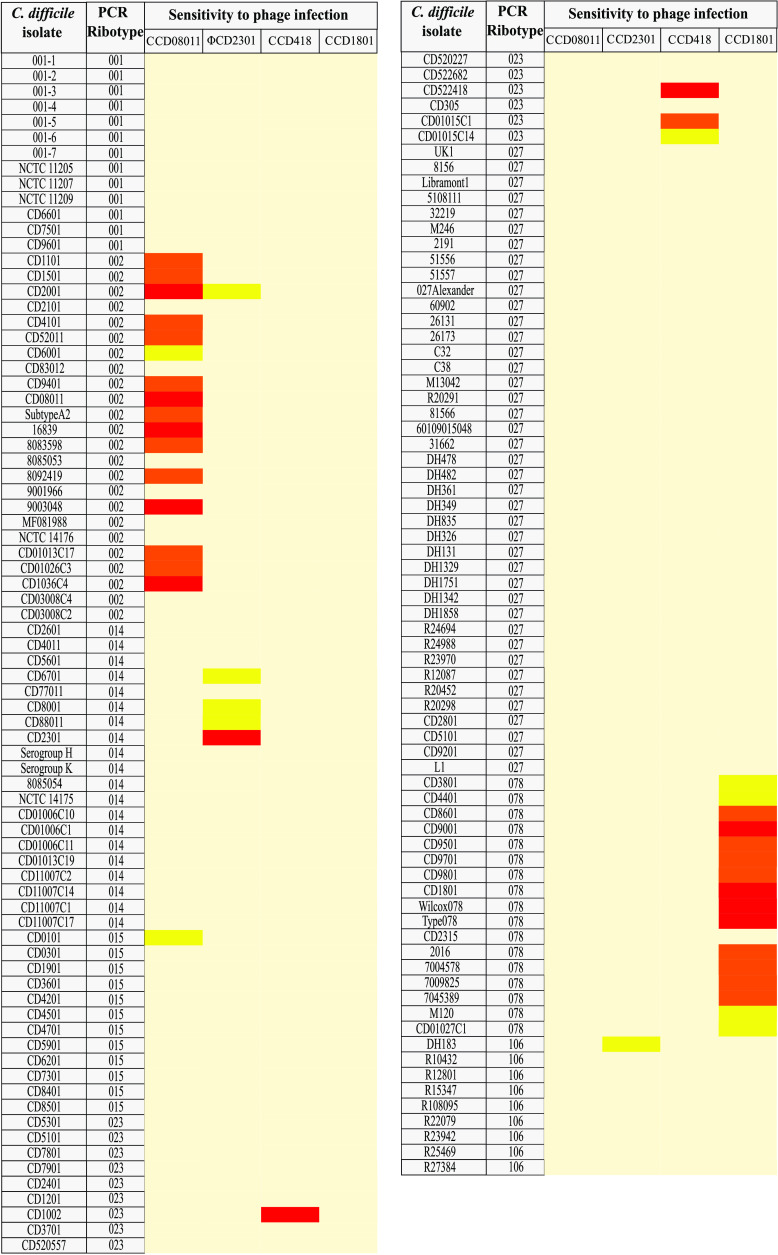
Heat map of C. difficile sensitivity to bacteriophage infection. Phage host range was determined using standard plaque assays for phages ΦCD08011, ΦCD2301, ΦCD418, and ΦCD1801. Efficiency of plating (EOP) values are depicted for each infection study represented by color. Magnolia, resistant strain; yellow, EOP of <0.1; orange, EOP 0.1 to 1; red, EOP of >1.

To determine the host range coverage of our novel phages, we adopted a standard double agar overlay plaque assay (see Materials and Methods) on 162 clinical isolates of C. difficile of various RTs. This analysis revealed that ΦCD1801 had broad host range activity toward RT 078. Infection was observed for 15/16 isolates with various efficiencies of plating (see experimental), representing 93.8% coverage (Fig. 3). To our knowledge, these data indicate that ΦCD1801 has the greatest reported host range coverage within RT 078. To ascertain why our phage was unable to infect the resistant strain (CD2315), we analyzed the bacterial genome sequence (accession CP068554.1). Analysis using the PHASTER web tool (18) identified the presence of an intact prophage. Further inspection by means of EMBOSS pairwise sequence analysis (17) uncovered 100% nucleotide identity between the lysogenic repressor protein of ΦCD1801 and the prophage contained within the genome of CD2315. Given our later focus on the importance of *slpA* allele for phage infection, we blasted the *slpA*, *secA2*, and *cwp66* genes from CD2315 (accession CP068554.1) against the genome sequence for the archetypal strain M120 (accession FN665653.1), which is known to possess S-layer cassette (SLC) H2/6 (9). We found 100% nucleotide conservation for all three genes, suggesting that this isolate also possesses the H2/6 SLC as predicted (Fig. S1 to S3). Taken together, it is likely that the presence of an identical repressor protein is responsible for the lysogenic immunity of CD2315 toward phage infection by ΦCD1801.

In a manner similar to that described above, ΦCD08011 possessed a stringent affinity toward the RT from which it was isolated. Indeed, the phage could infect 15/23 RT 002 isolates representing 65.2% coverage (Fig. 3). The remaining two phages demonstrated host range activity remarkably lower than that of the above-mentioned phages. ΦCD2301 was able to infect only 3/19 RT 014 strains, representing only 15.8% host range coverage. In addition to the three RT 014 strains, ΦCD2301 was shown to infect the RT 106 isolate CDDH183, albeit with a low efficiency of plating (EOP) value. Finally, ΦCD418 was able to infect only 3/14 of the tested RT 023 strains, representing 21.4% host range coverage.

Taken together, it appears that the phages isolated herein appear to have essentially strict sensitivity to one particular RT. This differs from most of the previously published phages in which cross-ribotype sensitivity is frequently observed (15). This phenomenon is unexplained at present but could relate to the sample origin for phage isolation. In our study, we used sewage samples from the United Kingdom which are ultimately derived from human feces. Other studies utilized purely environmental samples for phage isolation, for example soil (15). It could also relate to our library of tested strains. Although we have tested a very large library of strains, our study was limited to only eight RTs. Consequently, it is possible that each phage is able to infect other RTs not tested in this study.

### Probing the S-layer as a surface receptor candidate for ΦCD1801.

The S-layer of C. difficile is a paracrystalline protein that coats the entire bacterial cell, comprised of a precursor protein SlpA that is posttranslationally cleaved into high-molecular-weight and low-molecular-weight SlpA derivatives (19). The gene encoding SlpA is located within a hypervariable S-layer cassette (SLC) comprising a five-gene cluster containing *slpA*, *sec2A*, *cwp2*, *cwp66*, and *cwp2790*. Thus far, 14 S-layer cassette types (SLCT) have been assigned, where the variability is ascribed mainly to sequence differences within the low-molecular-weight component of *slpA* (20). Research conducted on a novel R-type bacteriocin, coined Avidocin-CD, uncovered SlpA as the surface receptor for these novel antibacterial agents by identifying resistant mutants that had lost their S-layer (9); thereafter, they showed that cross-sensitivity could be conferred in an SLCT-dependent manner by switching the phage-derived RBP (9). The genome of the Avidocin was modified to include the gene encoding a predicted RBP mined from RT 027 myovirus prophage genomes and, as such, was capable of broad RT 027 killing (21). Taken together, these earlier works provide a clear indication that the S-layer is the likely receptor for myovirus infection of C. difficile. In support of this hypothesis, Phothichaisri and colleagues have suggested that a physical interaction occurs between phage particles and SlpA by means of native-PAGE analysis where addition of phage particles led to retardation during the electrophoresis (10). We wanted to build upon the above-mentioned findings by probing the ability for exogenous SlpA to permit cross-ribotype binding of phages to C. difficile.

**FIG 4:**
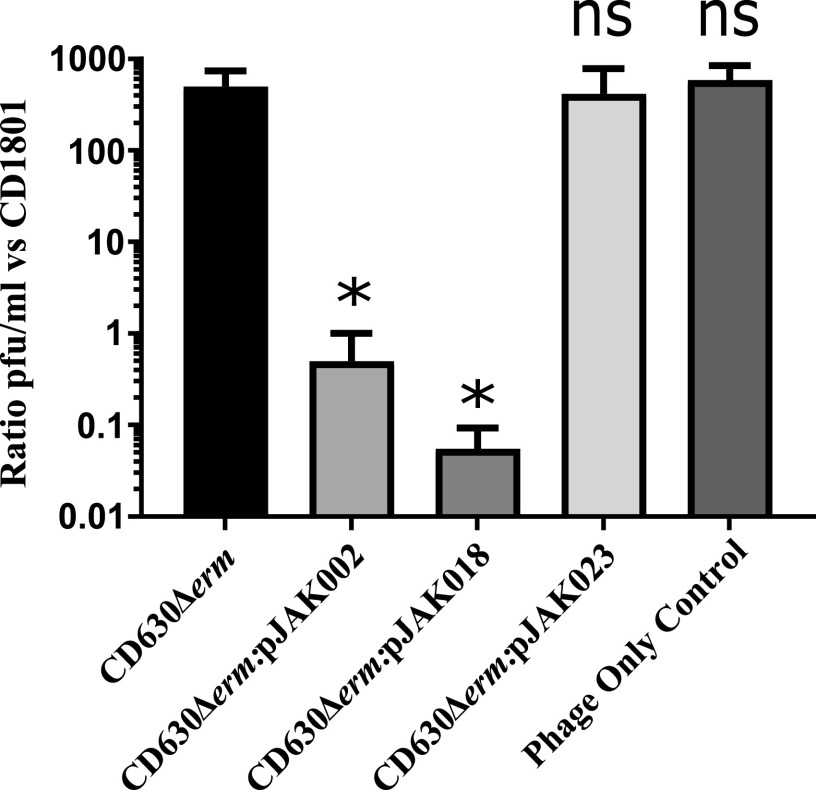
Phage binding is SLCT dependent in RT 012. The capacity for CD630 to bind to ΦCD1801 was assessed by means of a phage-binding assay with wild-type CD630 and CD630 harnessing plasmid-borne SLC H2/6 (pJK002), SLC 6 (pJK018), and SLC 2 (pJK023) under the control of a tetracycline inducible promoter. Following coincubation with wild-type or plasmid-bearing CD630, the titer of free ΦCD1801 particles was assessed through a plaque assay using the propagating strain CD18101 as an indicator. Data represent the mean ± SD of 3 biological replicates *, *P* < 0.05 according to one-way analysis of variance (ANOVA) followed by Dunnett’s multiple-comparison test.

In order to determine the relationship between various SLCTs and our bacteriophage, we conjugated pJAK002 comprising pRPF185, expressing the RT 078-derived hybrid S-layer cassette (H2/6), as well as SLCT 2 (pJAK023) and SLCT 6 (pJAK018) (9), into the RT 012 strain CD630 (SLCT 7). Following successful conjugation, the recipient strain was assessed for its ability to bind ΦCD1801 by means of a binding assay. These analyses revealed that CD630 was unable to bind ΦCD1801 (Fig. 4). Thus, no substantial reduction in titer was observed (≥1-log) between the initial phage inoculum and the number of unbound phage particles following plaque assay in strain CD1801. However, in the presence of plasmid-borne SLCs H2/6 and 6 encoded on the plasmids pJAK002 and pJAK018, respectively, ΦCD1801 bound to CD630 as indicated by a substantial decrease in the number of unbound phage particles observed (Fig. 4). The typical RT 078 S-layer cassette is considered hybrid in nature since its *cwp66* component is homologous to SLCT 2 while the *slpA* and *secA2* components are more similar to SLCT 6 (22). Accordingly, ΦCD1801 bound to SLCT 6 and H2/6 but not to SLCT 2, for which the *slpA* component is genetically distinct (Fig. 4). Taken together, these data demonstrate that phage binding for ΦCD1801 is dependent on the SLCT. Therefore, our results corroborate the above-mentioned findings (9, 10), providing further evidence to suggest that SlpA is the likely surface receptor for bacteriophage infection by C. difficile myoviruses. Without demonstrating the expression of exogenous SlpA, we cannot definitively state that our results are solely a consequence of the intended effects of plasmid-borne *slpA*, despite the inclusion of appropriate experimental controls. Indirect effects on the cell surface cannot be excluded.

Large-scale phylogenetic analysis of C. difficile whole-genome sequences has uncovered that sequence typing (ST) is a poor indicator of SLCT, since strains belonging to certain STs can possess essentially random SLCTs (22). PCR ribotyping appears to give a much stronger correlation. This is particularly true for RT 078, where analysis of whole-genome sequencing (WGS) revealed full congruence between 40 RT 078 strains with SLCT H2/6 (23). However, certain RTs have been shown to be divergent with regard to their SLCT, for example RT 012 (SLCT 7 and 8), RT 014 (SLCT 6 and 10), RT 015 (SLCT 4 and 9), and RT 126 (SLCT H2/6, 8 and unassigned DK1) (9, 23). As such, future studies should be concerned with not only RT but also SLCT when assessing the host range activity of C. difficile phages.

Given the notion that 14 confirmed SLCTs exist for C. difficile (cassette types 1 to 13 and the hybrid cassette H2/6), in addition to the unassigned cassette DK1 found in a subset of RT 126 strains (23), it seems sensible to direct research attention toward the isolation of phages, individually possessing broad host range activity toward each of these. Doing so has the potential to considerably enhance the therapeutic prospect of phage therapy for the treatment of CDI.

**Conclusions.** Four novel bacteriophages infecting C. difficile have been isolated from United Kingdom sewage samples: ΦCD08011, ΦCD418, ΦCD1801, and ΦCD2301. While ΦCD08011, ΦCD418, ΦCD1801, and ΦCD2301 were comparable to other phages reported in the literature, ΦCD1801 was shown to possess the broadest reported host range activity toward RT 078 strains of C. difficile, wherein 15/16 (93.75%) of clinical isolates were susceptible to lysogenic infection. To corroborate previous suggestions that the S-layer is important for myovirus infection, our phage-binding assay data indicated that ΦCD1801 was unable to bind to the RT 012 strain CD630 (SLCT 7). However, harboring plasmid-borne *slpA* alleles corresponding to the RT 078-SLCT (H2/6 and SLCT 6) was able to remedy this phenomenon. Consequently, these data provide supporting evidence to previous studies which suggest that the S-layer constituent, SlpA, represents the likely phage receptor on the surface of C. difficile.

## MATERIALS AND METHODS

### Routine growth of C. difficile strains.

C. difficile isolates were routinely grown on brain heart infusion (BHI) medium supplemented with 0.5% yeast extract, 0.1% l-cysteine, and C. difficile selective supplement comprising 250 µg/mL d-cycloserine and 8 µg/mL cefoxitin (Oxoid, USA), referred to as BHIs. Strains were maintained at 37°C under anaerobic conditions in a Don Whitley anaerobic workstation (80% N_2_, 10% CO_2_, and 10% H_2_).

### Isolation of C. difficile from stool samples.

Novel C. difficile isolates used within this study were isolated from patient fecal samples collected at the Queens Medical Centre, Nottingham, UK. Stool samples were homogenized 1:1 with phosphate-buffered saline (PBS), heat shocked at 80°C for 15 min, and centrifuged for 5 min at 1500 × *g*. A 50-μL aliquot of the supernatant was used to inoculate triplicate cycloserine cefoxitin egg yolk (CCEY) agar plates (LabM, UK) in an anaerobic cabinet and incubated for 48 h. Plates were prepared by autoclaving 48 g premixed CCEY in 1 L distilled water (dH_2_O) and adding 40 mL (4%) egg yolk emulsion (Lab M, UK) post-autoclave. Prior to use, the plates were kept under anaerobic conditions for a minimum of 4 h. Putative C. difficile isolates were transferred into a 96-microtiter plate containing 200 μL BHIs broth, one per well and up to 20 per patient sample. Microtiter plates were sealed with breathable sterile film and incubated overnight in anaerobic conditions. A separate 96-well microtiter plate contained 180 μL PCR-grade H_2_O where a 1:10 dilution was made from the overnight broth cultures. A drop of glycerol was then added to the broth cultures and resealed using fresh breathable sterile film and stored at −80°C. The H_2_O culture mix was covered in breathable sterile film and stored at −20°C for subsequent use as a PCR template for ribotyping. A complete list of strains used in this study is provided in Table S1.

### Ribotyping.

Ribotyping of the clinical isolates was performed exactly as described previously (24), following the extraction of DNA from the above-mentioned treated stool samples by heating at 95°C for 20 min after initial defrosting. PCR products were visualized using a Qiaexcel using the Qiaexcel DNA High Resolution (Qiagen, Germany). Band profiles were analyzed by eye in the first instance before each isolate was sent to the C. difficile Reference Network (CDRN) at Leeds Royal Infirmary (Leeds, UK) for official assignment of strain ribotype.

### Isolation of phages.

Sewage samples were obtained from an anaerobic digester at Stoke Bardolph sewage treatment plant in Nottinghamshire, United Kingdom. The sewage sample (50 mL) was enriched overnight, anaerobically, with the dry components of BHIs with the addition of 1% taurocholate and MgCl_2_. Subsequently, the enrichment cultures were centrifuged at 10,000 × *g* for 10 min at 4°C to remove bacteria and debris. The supernatant was filter sterilized (0.22 µM filter, Millipore) and stored at 4°C. Potential RT 078 hosts were selected from isolates obtained at the Queens Medical Centre, Nottingham, United Kingdom. Phages were identified through plaque formation, and plaques were subsequently purified three times. Lysogens of the isolated phage within the propagating strain CD1801 were isolated using the spot on the plate method as described previously (25). Briefly, high-titer phage stocks (10^8^ PFU/mL) were spotted over a confluent layer of C. difficile following overnight incubation on 0.5% (wt/vol) BHIs top agar plates. Five colonies growing within the zone of clearing were restreaked to purity three times on BHIs agar plates. Lysogens were then confirmed by induction of prophage in accordance with established methods (26). Twenty milliliters of C. difficile overnight culture in BHIs broth was induced with of 3 µg/mL mitomycin C before incubating for a further 24 h at 37°C under anaerobic conditions. Thereafter, cells were pelleted by centrifugation at 3,400 × *g* for 10 min at 4°C and the supernatant was sterilized by passage through a 0.22-µm membrane filter. Filtered induced lysates were stored at 4°C and tested for the presence of phages by plaque assay. Finally, lysogens were screened for their immunity to further phage infection by means of plaque assay (see below).

### Enumeration of phages.

Phages were enumerated using the double agar overlay plaque assay, in accordance with a published protocol (27). Briefly, a 1% (vol/vol) inoculum of C. difficile overnight culture was transferred to 20 mL fresh prereduced BHIs broth and incubated anaerobically for 6 h to an optical density at 600 nm (OD_600_) value of 0.8 to 1.0. Thereafter, 1 mL of the resultant culture was mixed with 200 µL of sewage enrichment lysate or phage stock and 3 mL of 0.5% BHIs (wt/vol) top agar and poured over 1% (wt/vol) BHIs agar plates. Plates were incubated anaerobically overnight before determination of the presence of phages or enumeration of phage titer.

### Transmission electron microscopy.

Isolated phage lysates (>10^9^ PFU/mL) were precipitated by 1 M ammonium acetate (Sigma-Aldrich, USA) with centrifugation steps at 21,000 × *g* for 75 min. The precipitated phage particles were stained with 10 µL of 2% uranyl acetate (Sigma-Aldrich, USA) for 30 s on 200 mesh formvar carbon-coated copper grids before they were visualized through transmission electron microscopy (TEM) following an established method (14).

### Extraction of phage genomic DNA.

Phage genomic DNA was extracted from crude phage lysate using a modified phenol-chloroform method (8). A 2 mL volume of crude phage lysate (∼10^9^ PFU/mL) was mixed with 25 µL MgCl_2_ (1 M, Sigma), 0.8 µL DNase I (2,000 U/mL; Thermo Fisher Scientific), and 20 µL RNase A (10 mg/mL, Thermo Fisher Scientific) and incubated at room temperature for 30 min. Subsequently, 80 µL EDTA (0.5 M, Thermo Fisher Scientific), 5 µL proteinase K (20 mg/mL, Qiagen), and 100 µL 10% SDS (Thermo Fisher Scientific) were added to the phage-MgCl_2_ mixture and incubated at 55°C for 1 h. The resulting liquid was aliquoted into 4 phase lock tubes (Quanta Biosciences) and extracted 3 times with an equal volume of phenol/chloroform/isoamylalcohol (25:24:1, Sigma). A final extraction with an equal volume of chloroform (Sigma) was conducted before the DNA was precipitated using 2 volumes 100% ethanol and 0.1 volumes sodium acetate (Sigma) and incubated on ice for 5 min. All centrifugation steps were performed at 13,000 rpm for 5 min. The precipitated DNA was centrifuged at 13,000 rpm for 10 min, and the resulting pellet was washed with 1 mL 70% ethanol. The centrifugation step was repeated and the pellet was air-dried before the DNA was dissolved in 10 mM Tris-HCl (pH 8.5, Qiagen) at 65°C for 20 min. Eluted DNA was pooled, quantified using NanoDrop Lite spectrophotometer (Thermo Scientific), and stored at 4°C before sequencing.

### Sequencing and annotation of genomes.

Whole-genome sequencing of C. difficile strain CD2315 and purified phages was conducted by DeepSeq (University of Nottingham) using an Illumina MiSeq platform. For CD2315, paired reads were aligned to the archetypal genome sequence of strain M120. For the phage genomes, raw sequencing reads were assembled into a single contig using *de novo* assembly function within CLC Genomics Workbench 9.5.3 (Qiagen). Artemis software (28) was used to identify putative open reading frames (ORFs). Manual genome annotation was completed using NCBI BLASTp, UniProt, and pfam databases to assign putative protein functions. ORFs were manually trimmed to the correct start codon based on the presence of ribosome binding sites and promoter sequences.

### Determination of phage host range testing.

Standard plaque assay was used to determine the host range of the isolated phage using an ∼109 PFU/mL stock according to established methods (27). Efficiency of plating was determined for each indicator strain by comparison of the phage titer using the propagating strain against the phage titer using the indicator strain (EOP = phage titer of propagating strain/phage titer of indicator strain). Tested strains are listed in Table S1. PHAge Search Tool Enhanced Release (PHASTER) was used to identify prophage regions within the genome of resistant isolates (18). Putative repressor proteins were aligned using The European Molecular Biology Open Software Suite (EMBOSS) pairwise alignment tool (16).

### S-layer receptor testing.

pRPF185 plasmids expressing the hybrid RT 078 S-layer cassette (H2/6) (pJAK002) and the individual SLCs 2 (pJAK023) and 6 (pJAK018), under the control of an anhydrotetracycline-inducible promoter, were obtained from Robert Fagan (University of Sheffield, UK) (29). pRPF185 was conjugated into C. difficile 630 exactly as described previously using Escherichia coli CA434 as a conjugal donor strain (30). A 1% inoculum of an overnight culture of the transformed strain was transferred to 20 mL prereduced BHIs broth and incubated for 4 h before being induced with anhydrotetracycline (Sigma-Aldrich, USA) to a final concentration of 500 ng/mL in a 20 mL culture for 1 h under anaerobic conditions. To detect phage binding in the presence and absence of the RT 078 S-layer cassette, a binding assay was conducted. Therein, 20 mL of induced cultures was harvested by centrifugation and the resulting cell pellet was resuspended in 10 µL of phage (10^4^ PFU/mL). This was incubated for 15 min under anaerobic conditions to allow the phage to bind before being resuspended in 1 mL BHIs broth. A final centrifugation step was completed to remove the bacterial cells. The number of phage particles in the supernatant that had not bound to cells was enumerated using plaque assay as mentioned above, using CD1801 as an indicator. A substantial reduction in phage titer from the infection phage titer is indicative of phage binding. C. difficile 630 and 1801 were used as negative and positive binding controls, respectively.
