# Supplementary material for: A Novel Bacteriophage with Broad Host Range against Clostridioides difficile Ribotype 078 Supports SlpA as the Likely Phage Receptor
Source: Microbiol Spectr. 2022 Feb 2;10(1):e02295-21. doi: 10.1128/spectrum.02295-21 (PMC8809339; doi:10.1128/spectrum.02295-21)
Supplement: SUPPLEMENTAL FILE 1 — Supplemental material. Download SPECTRUM02295-21_Supp_1_seq8.pdf, PDF file, 0.5 MB [file spectrum02295-21_supp_1_seq8.pdf]

Supplementary information

A novel bacteriophage with broad host-range against *Clostridioides difficile* ribotype 078 supports SlpA as the likely phage receptor

M.J Whittle [1,2], T.W Bilverstone [1,2], R.J van Esveld[3], A-C. Lücke[4], M.M Lister [1,2], S.A Kuehne [1,2] and N.P Minton[1,2]*

[1] Clostridia Research Group, BBSRC/EPSRC Synthetic Biology Research Centre (SBRC), School of Life Sciences, Biodiscovery Institute, The University of Nottingham, Nottingham, NG7 2RD, UK.

[2] NIHR Nottingham Biomedical Research Centre, Nottingham University Hospitals NHS Trust and the University of Nottingham, Nottingham, NG7 2RD, UK.

[3]Faculty of Medicine, Leiden University Medical Centre, the Netherlands.

[4] Hannover Medical School, Carl-Neuberg-Straße 1, 30625 Hannover, Germany

Corresponding author: nigel.minton@nottingham.ac.uk

Keywords: Bacteriophage, phage therapy, *Clostridioides difficile* (*Clostridium difficile*), S-layer, SlpA.

Table S1: *C. difficile* isolates used in this study.

C. difficile isolate	PCR ribotype	Origin	Source/Reference
001-1	RT001	Netherlands	Ed Kujiper
001-2	RT001	Netherlands	Ed Kujiper
001-3	RT001	Netherlands	Ed Kujiper
001-4	RT001	Netherlands	Ed Kujiper
001-5	RT001	Netherlands	Ed Kujiper
001-6	RT001	Netherlands	Ed Kujiper
001-7	RT001	Netherlands	Ed Kujiper
NCTC 11205	RT001	Sheffield, UK	NCTC
NCTC 11207	RT001	Sheffield, UK	NCTC
NCTC 11209	RT001	Sheffield, UK	NCTC
CD66011	RT001	Nottinghamshire, UK	SBRC Nottingham
CD7501	RT001	Nottinghamshire, UK	SBRC Nottingham
CD9601	RT001	Nottinghamshire, UK	SBRC Nottingham
CD1101	RT002	Nottinghamshire, UK	SBRC Nottingham
CD1501	RT002	Nottinghamshire, UK	SBRC Nottingham
CD2001	RT002	Nottinghamshire, UK	SBRC Nottingham
CD2101	RT002	Nottinghamshire, UK	SBRC Nottingham
CD4101	RT002	Nottinghamshire, UK	SBRC Nottingham
CD52011	RT002	Nottinghamshire, UK	SBRC Nottingham
CD6001	RT002	Nottinghamshire, UK	SBRC Nottingham
CD83012	RT002	Nottinghamshire, UK	SBRC Nottingham
CD9401	RT002	Nottinghamshire, UK	SBRC Nottingham
CD08011	RT002	Nottinghamshire, UK	SBRC Nottingham
SubtypeA2	RT002	Netherlands	Ed Kujiper

16839	RT002	Hungary	Ed Kujiper
8083598	RT002	Netherlands	Ed Kujiper
8085053	RT002	Netherlands	Ed Kujiper
8092419	RT002	Netherlands	Ed Kujiper
9001966	RT002	Netherlands	Ed Kujiper
9003048	RT002	Netherlands	Ed Kujiper
MF081988	RT002	Ireland	Ed Kujiper
TL178 (NCTC 14176)	RT002	Northern Ireland	NCTC
CD01013C17	RT002	Nottinghamshire, UK	SBRC Nottingham
CD01026C3	RT002	Nottinghamshire, UK	SBRC Nottingham
CD1036C4	RT002	Nottinghamshire, UK	SBRC Nottingham
CD03008C4	RT002	Nottinghamshire, UK	SBRC Nottingham
CD03008C2	RT002	Nottinghamshire, UK	SBRC Nottingham
CD2601	RT014	Nottinghamshire, UK	SBRC Nottingham
CD4011	RT014	Nottinghamshire, UK	SBRC Nottingham
CD5601	RT014	Nottinghamshire, UK	SBRC Nottingham
CD6701	RT014	Nottinghamshire, UK	SBRC Nottingham
CD77011	RT014	Nottinghamshire, UK	SBRC Nottingham
CD8001	RT014	Nottinghamshire, UK	SBRC Nottingham
CD88011	RT014	Nottinghamshire, UK	SBRC Nottingham
CD2301	RT014	Nottinghamshire, UK	SBRC Nottingham
serogroup H	RT014	Netherlands	Ed Kujiper
serogroup K	RT014	Netherlands	Ed Kujiper
8085054	RT014	Netherlands	Ed Kujiper
TL176 (NCTC 14175)	RT014	Cambridge	NCTC
CD01006C10	RT014	Nottinghamshire, UK	SBRC Nottingham

CD01006C1	RT014	Nottinghamshire, UK	SBRC Nottingham
CD01006C11	RT014	Nottinghamshire, UK	SBRC Nottingham
CD01013C19	RT014	Nottinghamshire, UK	SBRC Nottingham
CD11007C2	RT014	Nottinghamshire, UK	SBRC Nottingham
CD11007C14	RT014	Nottinghamshire, UK	SBRC Nottingham
CD11007C1	RT014	Nottinghamshire, UK	SBRC Nottingham
CD11007C17	RT014	Nottinghamshire, UK	SBRC Nottingham
CD0101	RT015	Nottinghamshire, UK	SBRC Nottingham
CD0301	RT015	Nottinghamshire, UK	SBRC Nottingham
CD1901	RT015	Nottinghamshire, UK	SBRC Nottingham
CD3601	RT015	Nottinghamshire, UK	SBRC Nottingham
CD4201	RT015	Nottinghamshire, UK	SBRC Nottingham
CD4501	RT015	Nottinghamshire, UK	SBRC Nottingham
CD5701	RT015	Nottinghamshire, UK	SBRC Nottingham
CD5901	RT015	Nottinghamshire, UK	SBRC Nottingham
CD6201	RT015	Nottinghamshire, UK	SBRC Nottingham
CD7301	RT015	Nottinghamshire, UK	SBRC Nottingham
CD8401	RT015	Nottinghamshire, UK	SBRC Nottingham
CD8501	RT015	Nottinghamshire, UK	SBRC Nottingham
CD5301	RT023	Nottinghamshire, UK	SBRC Nottingham
CD5101	RT023	Nottinghamshire, UK	SBRC Nottingham
CD7801	RT023	Nottinghamshire, UK	SBRC Nottingham
CD7901	RT023	Nottinghamshire, UK	SBRC Nottingham
CD2401	RT023	Nottinghamshire, UK	SBRC Nottingham
CD1202	RT023	Nottinghamshire, UK	SBRC Nottingham
CD1002	RT023	Nottinghamshire, UK	SBRC Nottingham
CD3701	RT023	Nottinghamshire, UK	SBRC Nottingham
CD520557	RT023	Nottinghamshire, UK	SBRC Nottingham

CD520227	RT023	Nottinghamshire, UK	SBRC Nottingham
CD522682	RT023	Nottinghamshire, UK	SBRC Nottingham
CD522418	RT023	Nottinghamshire, UK	SBRC Nottingham
CD305	RT023	London, UK	Brendan Wren
CD01015C1	RT023	Nottinghamshire, UK	SBRC Nottingham
CD01015C14	RT023	Nottinghamshire, UK	SBRC Nottingham
UK1	RT027	Stoke Mandeville, UK	Jon Brazier
L1	RT027	Unknown	Ed Kujiper
8156	RT027	France	Ed Kujiper
Libramont1	RT027	France	Ed Kujiper
5108111	RT027	Netherlands	Ed Kujiper
32219	RT027	Luxembourg	Ed Kujiper
M246	RT027	Ireland	Ed Kujiper
2191	RT027	Ireland	Ed Kujiper
51556	RT027	Germany	Ed Kujiper
51557	RT027	Germany	Ed Kujiper
027Alexander	RT027	Austria	Ed Kujiper
60902	RT027	Switzerland	Ed Kujiper
26131	RT027	Finland	Ed Kujiper
26173	RT027	Finland	Ed Kujiper
C32	RT027	USA	Ed Kujiper
C38	RT027	USA	Ed Kujiper
M13042	RT027	Canada	Ed Kujiper
R20291	RT027	Stoke Mandeville, UK	Jon Brazier
81566	RT027	France	Ed Kujiper
60109015048	RT027	Netherlands	Ed Kujiper
31662	RT027	Netherlands	Ed Kujiper
DH478	RT027	Taunton, UK	Val Hall

Dh482	RT027	Oxford, UK	Val Hall
DH361	RT027	Lewisham, UK	Val Hall
DH349	RT027	Cambridge, UK	Val Hall
DH835	RT027	Birmingham, UK	Val Hall
DH326	RT027	Sheffield UK	Val Hall
DH131	RT027	Manchester, UK	Val Hall
DH1329	RT027	Coventry, UK	Val Hall
DH1751	RT027	Bradford, UK	Val Hall
DH1342	RT027	Macclesfield, UK	Val Hall
DH1858	RT027	Sunderland, UK	Val Hall
R24694	RT027	Haverfordwest, UK	Val Hall
R24988	RT027	Antrim, UK	Val Hall
R23970	RT027	Ireland	Val Hall
R12087	RT027	Unknown	Val Hall
R20452	RT027	Canada	Val Hall
R20298	RT027	USA	Val Hall
CD2801	RT027	Nottinghamshire, UK	SBRC Nottingham
CD5101	RT027	Nottinghamshire, UK	SBRC Nottingham
CD9201	RT027	Nottinghamshire, UK	SBRC Nottingham
CD3801	RT078	Nottinghamshire, UK	SBRC Nottingham
CD4401	RT078	Nottinghamshire, UK	SBRC Nottingham
CD8601	RT078	Nottinghamshire, UK	SBRC Nottingham
CD9001	RT078	Nottinghamshire, UK	SBRC Nottingham
CD9501	RT078	Nottinghamshire, UK	SBRC Nottingham
CD9701	RT078	Nottinghamshire, UK	SBRC Nottingham
CD9801	RT078	Nottinghamshire, UK	SBRC Nottingham
CD1801	RT078	Nottinghamshire, UK	SBRC Nottingham
Wilcox078	RT078		Mark Wilcox

Type078	RT078	Leeds, UK	Ed Kujiper
CD2315	RT078	Hungary	Ed Kujiper
2016	RT078	Ireland	Ed Kujiper
7004578	RT078	Netherlands	Ed Kujiper
7009825	RT078	Netherlands	Ed Kujiper
7045389	RT078	Netherlands	Ed Kujiper
M120	RT078	Ireland	Brendan Wren
CD01027C1	RT078	Unknown	Nottingham
DH183	RT106	Leicester, UK	Val Hall
R10432	RT106	Bristol, UK	Val Hall
R12801	RT106	Bristol, UK	Val Hall
R15347	RT106	London, UK	Val Hall
R108095	RT106	Poole, UK	Val hall
R22079	RT106	Sheffield, UK	Val Hall
R23942	RT106	Cambridge, UK	Val Hall
R25469	RT106	Northern Ireland	Val Hall
R27384	RT106	Merthyr Tydfil, UK	Val Hall

Table S2: List of primers for the closure of phage genomes

Primer	Sequence 5'-3'-	Description (nucleotide position)
CD08011 *parA1*	CGTATTAAGGCTTGTACTC	Genome closure for Φ08011 (20781-20799)
CD08011 *pol1*	GCAAGAGCTGTTCATCAG	Genome closure for Φ08011 (22208-22225)
CD1801 *portal1*	GCTGATATAGCTAGAAGACAAG	Genome closure for Φ1801 (1469-1490)
CD1801 *portal2*	GACTTAGTTGCTATATCTGC	Genome closure for Φ1801 (3297-3316)
CD418 *phage protein 1*	GGTCACATATAATTCTATTTG	Genome closure for Φ418 (24970-24990)
CD418 *collar 1*	CGATTGATGAAGTTGTTAG	Genome closure for Φ418 (22851-22869)

Fig S1: Nucleotide sequence alignment for CD2315 and M120 *slpA*

Page 1 M120 slpA
1 82
CD2315 slpA
atgaataagaaaaatttagcaatggctatggctgctgttacagtagtaggttcggctgcaccagtatttgcagca
gatgaac
M120 slpA
atgaataagaaaaatttagcaatggctatggctgctgttacagtagtaggttcggctgcaccagtatttgcagca
gatgaac
....
....
83 164
CD2315 slpA
aagttaaataccaaaacacatatactgtagtgcaaagtaaatatgaaaaagctttaaaagatatgcaaaaggga
ttacaga
M120 slpA
aagttaaataccaaaacacatatactgtagtgcaaagtaaatatgaaaaagctttaaaagatatgcaaaaggga
ttacaga
....
....
165 246
CD2315 slpA
taaaaagataaaatcaatagcaatatcttatgaaggtaagccagttactactattactgttgcagatatggatac
aaaagga
M120 slpA
taaaaagataaaatcaatagcaatatcttatgaaggtaagccagttactactattactgttgcagatatggatac
aaaagga
....
....
247 328
CD2315 slpA
aaaacttcaacaaaagaagaattagcaagtgctttattaaagactactgttaatgataagttagataatttgggt
gatggag
M120 slpA
aaaacttcaacaaaagaagaattagcaagtgctttattaaagactactgttaatgataagttagataatttgggt
gatggag
....
....
329 410
CD2315 slpA
attatgtagattttgatataacttatgttggtgatgctgatagacttactgcaggggatcttaatacttttgcaa
aaggtat
M120 slpA
attatgtagattttgatataacttatgttggtgatgctgatagacttactgcaggggatcttaatacttttgcaa
aaggtat
....
....
411 492
CD2315 slpA
agcagatagtactgaaaagaaaattcctgctgctaaaggttctaactatggagttgctaaaacaaattctggaac
tggaaaa
M120 slpA
agcagatagtactgaaaagaaaattcctgctgctaaaggttctaactatggagttgctaaaacaaattctggaac
tggaaaa
....
....
493 574
CD2315 slpA
cttactacagatacagaggcagttatatctacttcaatagaaggtaaagttgagggaaataatcttacaataagt
cttaagg

M120 slpA
cttactacagatacagaggcagttatatctacttcaatagaaggtaaagttgagggaaataatcttacaataagt
cttaagg
....
....
575 656
CD2315 slpA
atgctccaagtaaagttggtgtaataggtgctaataatgatacacttgcagatgttacatttgcagatgatgcaa
aattaac
M120 slpA
atgctccaagtaaagttggtgtaataggtgctaataatgatacacttgcagatgttacatttgcagatgatgcaa
aattaac
....
....
657 738
CD2315 slpA
tgtatcagttggagatccaaaaatagatttagcaaaatcatttattttgatactaaaactggtaaattaggtgg
aatagta
M120 slpA
tgtatcagttggagatccaaaaatagatttagcaaaatcatttattttgatactaaaactggtaaattaggtgg
aatagta
....
....
739 820
CD2315 slpA
gaaaaagaaaatgatgcaactgaacatgcatatgtaagagtaataaatgctaaagaacaaacaatagatttagat
gctagtt
M120 slpA
gaaaaagaaaatgatgcaactgaacatgcatatgtaagagtaataaatgctaaagaacaaacaatagatttagat
gctagtt
....
....
821 902
CD2315 slpA
catataaatcagctgaagatttagctaaagcttatgcatttgatgtaaatgagcttaagactctttacactgaaa
tagaagc
M120 slpA
catataaatcagctgaagatttagctaaagcttatgcatttgatgtaaatgagcttaagactctttacactgaaa
tagaagc
....
....
Page 2 M120 slpA
903 984
CD2315 slpA
atatcaaaaagatagtaataataaaactgataaagttcaaatagttgatggtaaatatcaaacaatactttatgc
tgaagga
M120 slpA
atatcaaaaagatagtaataataaaactgataaagttcaaatagttgatggtaaatatcaaacaatactttatgc
tgaagga
....
....
985 1066
CD2315 slpA
aagagattaactactaaatcagcaactcaagcttctaaattagcagatgaaaactcaccacttaagttagttata
aaagcag
M120 slpA
aagagattaactactaaatcagcaactcaagcttctaaattagcagatgaaaactcaccacttaagttagttata
aaagcag
....
....
1067 1148

CD2315 slpA
ataaattaaaagatttaaaggattatgttgaagatttaagaaatgctaataatggttactcaaatactgttactg
tagcagg
M120 slpA
ataaattaaaagatttaaaggattatgttgaagatttaagaaatgctaataatggttactcaaatactgttactg
tagcagg
....
....
1149 1230
CD2315 slpA
tgatgatagaatagaaactgcaatagaattaagtagtaaatactataataattcagatgaagacaatgctataac
agaagat
M120 slpA
tgatgatagaatagaaactgcaatagaattaagtagtaaatactataataattcagatgaagacaatgctataac
agaagat
....
....
1231 1312
CD2315 slpA
gctgttaataatgttgtattagttggatctcaagcaatagttgatggacttgttgcatcacctttagcttcagag
aagaaag
M120 slpA
gctgttaataatgttgtattagttggatctcaagcaatagttgatggacttgttgcatcacctttagcttcagag
aagaaag
....
....
1313 1394
CD2315 slpA
ctcctttattattaacttcaaaagataaattagattcaaatgtaaaatctgaaattaaaagagttatgaatttaa
aaactac
M120 slpA
ctcctttattattaacttcaaaagataaattagattcaaatgtaaaatctgaaattaaaagagttatgaatttaa
aaactac
....
....
1395 1476
CD2315 slpA
aacaggaataaacaattctaagaaagtttacctagctggtggagttaattctatatctaaagaagttgaaaatga
attaaaa
M120 slpA
aacaggaataaacaattctaagaaagtttacctagctggtggagttaattctatatctaaagaagttgaaaatga
attaaaa
....
....
1477 1558
CD2315 slpA
gatatgggacttaaagtaacaagattatcaggagatgatagatatgctacttcattagaaatagctgatgaaata
ggtttag
M120 slpA
gatatgggacttaaagtaacaagattatcaggagatgatagatatgctacttcattagaaatagctgatgaaata
ggtttag
....
....
1559 1640
CD2315 slpA
atgatgataaagcattcgtagtaggtggaacaggacttgcagatgctatgagtatagctccagttgcttctcaat
taaatga
M120 slpA
atgatgataaagcattcgtagtaggtggaacaggacttgcagatgctatgagtatagctccagttgcttctcaat
taaatga

....
....
1641 1722
CD2315 slpA
aaaaggtgatgctacaccaatagttgtagttgatggaaaagctaaagaattaagctcagcagctgaagatttctt
agatgat
M120 slpA
aaaaggtgatgctacaccaatagttgtagttgatggaaaagctaaagaattaagctcagcagctgaagatttctt
agatgat
....
....
1723 1804
CD2315 slpA
tcacaagttgatataataggtggtaaaaatagcgtttctaaagacatggaagatgctatagatgatgctacagga
aaatctc
M120 slpA
tcacaagttgatataataggtggtaaaaatagcgtttctaaagacatggaagatgctatagatgatgctacagga
aaatctc
....
....
Page 3 M120 slpA
1805 1886
CD2315 slpA
caaatagagttagtggagatgatagacaagaaactaatgcagaagtattaaaagaatctgattatttcccagatg
gtgcagt
M120 slpA
caaatagagttagtggagatgatagacaagaaactaatgcagaagtattaaaagaatctgattatttcccagatg
gtgcagt
....
....
1887 1968
CD2315 slpA
aaattactttgttgctaaagatggatctacaaaagaagaccaattagttgatgcattagcagcagcaccagtagc
agccaac
M120 slpA
aaattactttgttgctaaagatggatctacaaaagaagaccaattagttgatgcattagcagcagcaccagtagc
agccaac
....
....
1969 2050
CD2315 slpA
tttggaagaacttataatataaaagataatgatagttctggaacagtttctccagcaccaatcatattagcaact
gattcat
M120 slpA
tttggaagaacttataatataaaagataatgatagttctggaacagtttctccagcaccaatcatattagcaact
gattcat
....
....
2051 2132
CD2315 slpA
tatcttcagatcaaaatgttgcaataagtaaagcattaccttctggaaaatcaggagataatttagtacaagttg
gtaaggg
M120 slpA
tatcttcagatcaaaatgttgcaataagtaaagcattaccttctggaaaatcaggagataatttagtacaagttg
gtaaggg
....
....
2133 2181
CD2315 slpA tatagcaaactcagtaatcactaagataaaagacttattagatatgtag
M120 slpA tatagcaaactcagtaatcactaagataaaagacttattagatatgtag

....
....

Fig S2: Nucleotide sequence alignment for CD2315 and M120 *secA2*

Page 1 M120 secA2
1 82
CD2315 secA2
atgtcagttttagatacaatacttgataaagcagatgaacaagaaattaagaagttgaatttaatagtagataaa
atagagg
M120 secA2
atgtcagttttagatacaatacttgataaagcagatgaacaagaaattaagaagttgaatttaatagtagataaa
atagagg
....
....
83 164
CD2315 secA2
ctttggaagaaaaaatacaaaacttatctgatgatgaattgaaagaaatgactaatgtatttaggaatagattaa
ataatgg
M120 secA2
ctttggaagaaaaaatacaaaacttatctgatgatgaattgaaagaaatgactaatgtatttaggaatagattaa
ataatgg
....
....
165 246
CD2315 secA2
agaaactttagatgacatattaatggaagcttttgcagttgtaagagaagtatcaaaaagaaaattaggaatgcg
tcaatat
M120 secA2
agaaactttagatgacatattaatggaagcttttgcagttgtaagagaagtatcaaaaagaaaattaggaatgcg
tcaatat
....
....
247 328
CD2315 secA2
agagtacagttaattggtgggatagtaatacatcaaggtaaaatcgctgagatgaaaacaggtgaaggtaaaacg
ttagttg
M120 secA2
agagtacagttaattggtgggatagtaatacatcaaggtaaaatcgctgagatgaaaacaggtgaaggtaaaacg
ttagttg
....
....
329 410
CD2315 secA2
aagtagcaccagtatatctaaatgctcttacaggtaagggtgtacatgtaatcacagtaaatgattacctggcag
aacgtga
M120 secA2
aagtagcaccagtatatctaaatgctcttacaggtaagggtgtacatgtaatcacagtaaatgattacctggcag
aacgtga
....
....
411 492
CD2315 secA2
taaagaacttatgagaccagtttatgaatctcttggtatgacagtaggagtaattatagctaaccaagaccaaaa
tataaga
M120 secA2
taaagaacttatgagaccagtttatgaatctcttggtatgacagtaggagtaattatagctaaccaagaccaaaa
tataaga

....
....
493 574
CD2315 secA2
aaacaacagtataaatgtgatataacttatggtacaaatagtgaatttggatttgattatttaagagataatatg
gttccag
M120 secA2
aaacaacagtataaatgtgatataacttatggtacaaatagtgaatttggatttgattatttaagagataatatg
gttccag
....
....
575 656
CD2315 secA2
atttatctcataaggtacaaagagaactaaactttgccatagtagatgaggtagactcaatattaatagatgaag
ctagaac
M120 secA2
atttatctcataaggtacaaagagaactaaactttgccatagtagatgaggtagactcaatattaatagatgaag
ctagaac
....
....
657 738
CD2315 secA2
tccgcttattattgcaggagatggagatgaagatttaaaactttatgaattggcaaatagctttataaaaactat
taaagaa
M120 secA2
tccgcttattattgcaggagatggagatgaagatttaaaactttatgaattggcaaatagctttataaaaactat
taaagaa
....
....
739 820
CD2315 secA2
gaagactttgagatggatagaaaagataaaactatagcattaacagcaagtggtataagcaaagctgagtcattt
tttggta
M120 secA2
gaagactttgagatggatagaaaagataaaactatagcattaacagcaagtggtataagcaaagctgagtcattt
tttggta
....
....
821 902
CD2315 secA2
taacaaaccttactgatataaagaatatagaattatatcatcacataaatcaagctttaagaggtcataagctta
tggaaaa
M120 secA2
taacaaaccttactgatataaagaatatagaattatatcatcacataaatcaagctttaagaggtcataagctta
tggaaaa
....
....
Page 2 M120 secA2
903 984
CD2315 secA2
agatgttgactatgttatctcaaatggagaagtaatgatagttgacgaatttacaggaagagtaatggatggtag
aagatat
M120 secA2
agatgttgactatgttatctcaaatggagaagtaatgatagttgacgaatttacaggaagagtaatggatggtag
aagatat
....
....
985 1066
CD2315 secA2
acagatggacttcaccaagctatagaagcaaaagaaggtgttgagataaataatgaatctaaaactatggctact
gtgactt

M120 secA2
acagatggacttcaccaagctatagaagcaaaagaaggtgttgagataaataatgaatctaaaactatggctact
gtgactt
....
....
1067 1148
CD2315 secA2
atcaaaatttcttcagactatatgaaaaactttctggtatgactggtactgcaaagacagaagaaggggaatttg
agtcaat
M120 secA2
atcaaaatttcttcagactatatgaaaaactttctggtatgactggtactgcaaagacagaagaaggggaatttg
agtcaat
....
....
1149 1230
CD2315 secA2
ctataaactaaatgttgttcaaataccaactaataaaccagtgattagagctgatttacatgataaggtatttaa
gacagaa
M120 secA2
ctataaactaaatgttgttcaaataccaactaataaaccagtgattagagctgatttacatgataaggtatttaa
gacagaa
....
....
1231 1312
CD2315 secA2
gaagaaaagtataatgctgttgtagaagaaataataaggatacataagactagacaaccaatacttgtaggaaca
gtttctg
M120 secA2
gaagaaaagtataatgctgttgtagaagaaataataaggatacataagactagacaaccaatacttgtaggaaca
gtttctg
....
....
1313 1394
CD2315 secA2
ttgaaaaatctgaaaaattatctagaatgcttaaaaaacaaggtattaagcatcaagtcttaaatgcaaaacaac
atgataa
M120 secA2
ttgaaaaatctgaaaaattatctagaatgcttaaaaaacaaggtattaagcatcaagtcttaaatgcaaaacaac
atgataa
....
....
1395 1476
CD2315 secA2
agaggcggagataatttctaaagctggtaaattagatgctataacgattgctacaaatatggcaggtagaggaac
ggatatt
M120 secA2
agaggcggagataatttctaaagctggtaaattagatgctataacgattgctacaaatatggcaggtagaggaac
ggatatt
....
....
1477 1558
CD2315 secA2
tctctaggtgcaggagatagagaagaagaacaacaagttaaaaatttaggtgggctttatgttataggaacagaa
agacacg
M120 secA2
tctctaggtgcaggagatagagaagaagaacaacaagttaaaaatttaggtgggctttatgttataggaacagaa
agacacg
....
....
1559 1640

CD2315 secA2
aatcaagaagaattgataatcagcttagaggacgttctggtcgtcaaggagacccaggtacatcaagattctttg
taagtct
M120 secA2
aatcaagaagaattgataatcagcttagaggacgttctggtcgtcaaggagacccaggtacatcaagattctttg
taagtct
....
....
1641 1722
CD2315 secA2
tgaagatgatgtaataaagctttatggtggaaaaactatagagaaacttatgaagagaacaagttcaaaggaaaa
tactgct
M120 secA2
tgaagatgatgtaataaagctttatggtggaaaaactatagagaaacttatgaagagaacaagttcaaaggaaaa
tactgct
....
....
1723 1804
CD2315 secA2
attgaaagtaaagcacttacaagagctatagaaagagctcaaaaaggtgtagaaggtaaaaattttgaaataaga
aaaaatg
M120 secA2
attgaaagtaaagcacttacaagagctatagaaagagctcaaaaaggtgtagaaggtaaaaattttgaaataaga
aaaaatg
....
....
Page 3 M120 secA2
1805 1886
CD2315 secA2
tccttaaatatgatgatactattaatgaacaaagaaaagttatatataatgaaagaaataaagttttaaataatg
aagacat
M120 secA2
tccttaaatatgatgatactattaatgaacaaagaaaagttatatataatgaaagaaataaagttttaaataatg
aagacat
....
....
1887 1968
CD2315 secA2
tcaagaagatattcaaaaaatggttaaagatatcatacaagaagcaggagaaaattatttagttggaagaaaaag
agattat
M120 secA2
tcaagaagatattcaaaaaatggttaaagatatcatacaagaagcaggagaaaattatttagttggaagaaaaag
agattat
....
....
1969 2050
CD2315 secA2
tatggatattttaaatatctatatagtacatttatgccagcagatacactattaatacctggagtagataaaaaa
agtgttc
M120 secA2
tatggatattttaaatatctatatagtacatttatgccagcagatacactattaatacctggagtagataaaaaa
agtgttc
....
....
2051 2132
CD2315 secA2
aagaaatagttgatagtacttatgaaatttcaaaaagagtttatgatttaaaaaagatgatgcttggtattgata
aggtttc
M120 secA2
aagaaatagttgatagtacttatgaaatttcaaaaagagtttatgatttaaaaaagatgatgcttggtattgata
aggtttc

....
....

2133 2214

CD2315 secA2

agagttggaaaagacagtacttttaaaagtagttgaccaatactggatagaccatatagatgctatggaacagtt
aagacag

M120 secA2

agagttggaaaagacagtacttttaaaagtagttgaccaatactggatagaccatatagatgctatggaacagtt
aagacag

....
....

2215 2296

CD2315 secA2

tatataggtcttaaatcttatgctcaaaaagacccgtttaaggaatatgctttagaaggatatgacatgtttgaa
gctttaa

M120 secA2

tatataggtcttaaatcttatgctcaaaaagacccgtttaaggaatatgctttagaaggatatgacatgtttgaa
gctttaa

....
....

2297 2346

CD2315 secA2 ataaaaatataagggaagcaacagtgcaatacttatataaatttaactaa

M120 secA2 ataaaaatataagggaagcaacagtgcaatacttatataaatttaactaa

....
....

Fig S3: Nucleotide sequence alignment for CD2315 and M120 *cwp66*

Page 1 M120 cwp66

1 82

CD2315 cwp66

atgaaaatatcaaaaaagatagtgtctttgttaactatgacatttttaactgttacattatatggaaatacatct
aatgcat

M120 cwp66

atgaaaatatcaaaaaagatagtgtctttgttaactatgacatttttaactgttacattatatggaaatacatct
aatgcat

....
....

83 164

CD2315 cwp66

ctacaaaagacacattaacaggttctggaagatgggaaacagcaataaaaataagtcaagctgggtggtcaaagt
ctgatac

M120 cwp66

ctacaaaagacacattaacaggttctggaagatgggaaacagcaataaaaataagtcaagctgggtggtcaaagt
ctgatac

....
....

165 246

CD2315 cwp66

agctgtattagtaaatgacaattccatagcagatgctttatcagctactccatttgcaaaagcaaaagatgcacc
aatatta

M120 cwp66

agctgtattagtaaatgacaattccatagcagatgctttatcagctactccatttgcaaaagcaaaagatgcacc
aatatta

....
....

247 328

CD2315 cwp66

ttaactcaaagtaataaattagatagtagaaccaaagcagaattaaaaagacttggtgtaaaaaatgtatattta
ataggag

M120 cwp66
ttaactcaaagtaataaattagatagtagaaccaaagcagaattaaaaagacttggtgtaaaaaatgtatattta
ataggag
....
....
329 410
CD2315 cwp66
gttcaattgcattaagttcagagattgaaaagcaattaaatgcggaaaatataaattttgaaagaatatctggaa
atagtag
M120 cwp66
gttcaattgcattaagttcagagattgaaaagcaattaaatgcggaaaatataaattttgaaagaatatctggaa
atagtag
....
....
411 492
CD2315 cwp66
atatgatacttctttaaaactagctgaaaagttaaataaggaaaaatctatctctaaaatagtagtagtaaatgg
agaaaag
M120 cwp66
atatgatacttctttaaaactagctgaaaagttaaataaggaaaaatctatctctaaaatagtagtagtaaatgg
agaaaag
....
....
493 574
CD2315 cwp66
ggacttgctgatgcagtaagtgttggagctatagctgctcaagaaaatatgccaataatactttctgattcagag
aatggaa
M120 cwp66
ggacttgctgatgcagtaagtgttggagctatagctgctcaagaaaatatgccaataatactttctgattcagag
aatggaa
....
....
575 656
CD2315 cwp66
ctgaagtagctgataattttatagatagtaaagatatagaaaaatcgtatgtaataggtggtacatattctattt
ctaattc
M120 cwp66
ctgaagtagctgataattttatagatagtaaagatatagaaaaatcgtatgtaataggtggtacatattctattt
ctaattc
....
....
657 738
CD2315 cwp66
tgtagaaagaaatttaccaaatgcaacaagaatagcaggcagtagtagaagtgaaacaaatgcaaagattataga
agaattt
M120 cwp66
tgtagaaagaaatttaccaaatgcaacaagaatagcaggcagtagtagaagtgaaacaaatgcaaagattataga
agaattt
....
....
739 820
CD2315 cwp66
tataaagatactgacataaaaaatatttatgttacaaaagatggtacaagaaataagaatgatttaatagattct
ttagcag
M120 cwp66
tataaagatactgacataaaaaatatttatgttacaaaagatggtacaagaaataagaatgatttaatagattct
ttagcag
....
....
821 902

CD2315 cwp66
tgggtgtattagcagctaaaaatagttctccaatagtattagcaggaaataagcttgatactactcaaaaagatg
tgttaaa
M120 cwp66
tgggtgtattagcagctaaaaatagttctccaatagtattagcaggaaataagcttgatactactcaaaaagatg
tgttaaa
....
....
Page 2 M120 cwp66
903 984
CD2315 cwp66
tactaaggttatagataaagttactcaaattggtggtttagggaatgaagatgctgtaaaaagcataatagatat
gcaagag
M120 cwp66
tactaaggttatagataaagttactcaaattggtggtttagggaatgaagatgctgtaaaaagcataatagatat
gcaagag
....
....
985 1066
CD2315 cwp66
aagactaaatatactatagaaactattgatgagttaaatgttgctataaaaaaggcagatgcaaatgatgtaata
atatttg
M120 cwp66
aagactaaatatactatagaaactattgatgagttaaatgttgctataaaaaaggcagatgcaaatgatgtaata
atatttg
....
....
1067 1148
CD2315 cwp66
aaccagagaaagatactagtataagtgattcattcaagatagaaacaaataaagctataactgtagaatttgatg
gagtatt
M120 cwp66
aaccagagaaagatactagtataagtgattcattcaagatagaaacaaataaagctataactgtagaatttgatg
gagtatt
....
....
1149 1230
CD2315 cwp66
taaaaaatctataactatagacatgcctaatggagatgtaaagaattttggagaaatttcagatgacataagaat
agataat
M120 cwp66
taaaaaatctataactatagacatgcctaatggagatgtaaagaattttggagaaatttcagatgacataagaat
agataat
....
....
1231 1312
CD2315 cwp66
ataaagaaaggtactttgattaatgaaggtagtatacaaggtatagatatttattctaagaatgggtgtaagata
gaaaata
M120 cwp66
ataaagaaaggtactttgattaatgaaggtagtatacaaggtatagatatttattctaagaatgggtgtaagata
gaaaata
....
....
1313 1394
CD2315 cwp66
caagtgatggagatatatggattataactatagatgctgatgctaaagatgtatatatagaaaatgatggagaca
taacaaa
M120 cwp66
caagtgatggagatatatggattataactatagatgctgatgctaaagatgtatatatagaaaatgatggagaca
taacaaa

....
....
1395 1476
CD2315 cwp66
aatatcaaataatgctccaggtgttataataaaaaattcaggaaagattgatttagtaaatggtaatgagcaacc
tgcaatt
M120 cwp66
aatatcaaataatgctccaggtgttataataaaaaattcaggaaagattgatttagtaaatggtaatgagcaacc
tgcaatt
....
....
1477 1558
CD2315 cwp66
agtggaaagaaaccaacaactaatgatactgaatataatgatgaaagagcgcgtggattgtctgttagtacaaag
ccttgct
M120 cwp66
agtggaaagaaaccaacaactaatgatactgaatataatgatgaaagagcgcgtggattgtctgttagtacaaag
ccttgct
....
....
1559 1640
CD2315 cwp66
caatacctgaaaaagatcgtgtaagagtgacgatatctagtgaacctaagagttcaagatataaaatttattata
gagttgt
M120 cwp66
caatacctgaaaaagatcgtgtaagagtgacgatatctagtgaacctaagagttcaagatataaaatttattata
gagttgt
....
....
1641 1722
CD2315 cwp66
agaagataaaccttctgctatgtatgtaggagaaaaaattagtgtaagaagttgggacttagcatcaaaatcaga
tggatct
M120 cwp66
agaagataaaccttctgctatgtatgtaggagaaaaaattagtgtaagaagttgggacttagcatcaaaatcaga
tggatct
....
....
1723 1804
CD2315 cwp66
tttgtagaaaaagctaaaaatggatcatatatagaagttgttgaaataaatacctcaactaataaagtttctaga
tggggaa
M120 cwp66
tttgtagaaaaagctaaaaatggatcatatatagaagttgttgaaataaatacctcaactaataaagtttctaga
tggggaa
....
....
Page 3 M120 cwp66
1805 1833
CD2315 cwp66 gatcaaatgtaactgatgatggattttaa
M120 cwp66 gatcaaatgtaactgatgatggattttaa
....
....
